# Crossing the Borders of Nanomedicine

**DOI:** 10.3390/ijms232415728

**Published:** 2022-12-12

**Authors:** Massimiliano Magro

**Affiliations:** Department of Comparative Biomedicine and Food Science, University of Padua, Viale dell’Università 16, 35020 Legnaro, Italy; massimiliano.magro@unipd.it; Tel.: +39-049-827263

Nanomedicine, being pressured by the increasing demands for fighting menacing diseases such as cancer, relies pragmatically on consolidated knowledge, namely on therapeutic strategies that are at an advanced stage of experimentation. Due to this traditional approach, innovative methodologies rooted in areas such as material sciences, biotechnology, and nanotechnology suffer from a strong competition. While nanomedicine expectations have raised the bar of excellence in terms of patient-tailored treatments, low patient discomfort and cost-effectiveness, what seems to be needed are smart nano-tools able to interplay with biological systems in a programmable way. Often, these smart multifunctional nanomaterials consist of an inorganic core and a series of organic and biological layers providing the biological activity (i.e., targeting, stimuli-responsiveness, and therapeutic effect). The big question is: How can a synthetic nanomaterial, comprising biological components and inorganic materials, be tailored with the specificity of a biological system and be programmed to a task in the human body? Starting at the very beginning of the story, when simple organic molecules evolved into ordered and functional biopolymers (i.e., metabolism-first hypothesis), naturally occurring minerals were already present on Earth crust. There is a large consensus in the belief that in the prebiotic era those inorganic materials were the mold where life was forged [1]. In my opinion, the idea of such a close biotic-abiotic relationship at the origin of life could still be inspirational to envisage a new generation of synthetic materials able to engage with complex biological pathways. Synthetic materials incorporating both biotic and abiotic parts could be intended as novel biological entities, recognized as self by cell receptors and by the immune system, to avoid triggering organism molecular defenses. The understanding and modeling of those factors affecting the mutual influences between biological and abiotic systems represents the common goal of several research areas spanning from prebiotic chemistry to early biotic systems biology, from bio-chiral selectivity to material science. Aiming at an actual introduction of nanotechnologies into the new era of medicine, it is of fundamental importance to avoid any waste of multidisciplinary knowledge, in order to gather hints from the whole mosaic of sophisticated biotic-abiotic interactions. The great body of information inherited from neighboring scientific fields, if neglected, would deprive nanomedicine of nuances that are not perceivable by keeping the attention focused solely on the medical field. The articles presented in the “Bio-Nano Interactions” collection, encompass topics such as biomineralization, cancer treatment and nanotoxicology, and embody the idea of crossing the borders of such disciplines. Specifically, selected nano-biomaterials and their medical applications are at the center of this collection and will be briefly presented in the next paragraphs. Among carbon nanomaterials, fluorescent carbon dots (CDs) have the potential as cell labelling agent as well as tools for anticancer treatment. CDs affinity towards cell organelles is dictated by their surface properties that affect the interaction with membranes and the distribution at the subcellular level. Havrdová et al. investigated the intracellular trafficking and cytotoxicity of CDs decorated with quaternary ammonium groups (QCDs) using two human cancer cell lines as models. The anticancer effect of QCDs in both cell lines resulted in the deformation of the cellular shape, mitotic catastrophe, restriction of movement and proliferation, as well as genotoxicity [2]. Therefore, aiming at anticancer treatment, the surface modification of CDs using quaternary ammonium groups can be viewed as a promising strategy. In a different study, the same group investigated QCD distribution inside two healthy mouse cell lines (i.e., NIH/3T3 and L929) by performing an overall cytotoxic and DNA damage analyses. Although QCDs penetration into nuclei was observed in both cases, NIH/3T3 did not show significant changes in viability nor DNA damage. Conversely, QCDs uptake in the nucleus of L929 was responsible of immediate apoptosis. With this in mind, the high permeability of QCDs to the nuclei and nucleoli remains an attracting feature of these nanoparticles. In addition, the intranuclear environment of NIH/3T3 cells influenced fluorescence properties of QCDs evoking a very interesting emission blue shift that was attributed to the formation of cationic QCDs and DNA complexes. Thus, as suggested by the Authors, new biological applications of QCDs can be envisaged, including DNA sensing. In the area of bio/inorganic composites and multilayered nanomaterials, Piosik and colleagues studied the effect of surface functionalization of magnetite nanoparticles using native and aminated starch, toward the process of cell internalization using model cell membranes. Amino groups attached to the starch in the outermost layer seemed to reduce, to some extent, their disruptive effect on the model membranes and improved the adsorption of the nanoparticles [3]. Biomineralization, the natural process whereby organisms biosynthesize mineralized tissues such as bones, dental structures, as well as mollusk shells, is a worthful example of the degree of complexity and specificity that can be achieved by the interaction between biological and inorganic surfaces [4]. Mineralized tissues are composed of hierarchically organized hydroxyapatite crystals, with a limited capacity to regenerate when demineralized or damaged past a critical level. Danesi et al. described the uniaxial hydroxyapatite growth on a nanoengineered amelogenin scaffold in combination with amelotin. This bio-inspired approach for hydroxyapatite growth study can be informative in deepening the comprehension of the molecular mechanism of hydroxyapatite formation in vitro as well as in understanding the mechanisms at play during mineralized tissue formation. This is of crucial importance for the development of artificial biomaterials capable of mineralized tissue regeneration [5]. The fibrillization of peptides is relevant to numerous diseases related to the deposition of amyloids. Although the formation of fibrils has been intensively studied, the role of surface topography support was mostly neglected. In this context, Hanke et al., investigated the aggregation of the type 2 diabetes-associated peptide hormone hIAPP in contact with flat or nanopatterned silicon oxide surfaces. Noteworthy, the nanopatterns led to retarded fibrillization and stronger amorphous aggregation, resulting in a lower propensity for nucleating amyloid fibrillization. The results demonstrate that nanoscale surface topography may modulate peptide and protein aggregation pathways in complex and intricate ways [6]. Despite its outstanding antimicrobial effect, the clinical application of low-molecular-weight organic ammonium has been limited by short-term functionality and high toxicity. However, polymerization of monomeric ammonium salts can be viewed as a bottom-up approach for developing a nano-sized entity with properties that can significantly differ from the individual molecular constituent. Schito et al. synthesized a water-soluble non-quaternary copolymeric ammonium salt (P7) by copolymerizing 2-methoxy-6-(4-vinylbenzyloxy)-benzylammonium hydrochloride monomer with N, N-di-methyl-acrylamide. Noteworthy, the so obtained P7, tested against several multidrug-resistant (MDR) clinical isolates of both Gram-positive and Gram-negative species, displaying a rapid non-lytic bactericidal activity. P7 can therefore represent a novel and potent tool capable of counteracting infections caused by bacteria that are resistant to the presently available antibiotics [7]. The two-edge sword of bio-promising nanotechnologies is their high efficiency at low concentrations that could represent potential organism-wide harmful effects. Beside the attracting properties of nanoparticles, it is crucial to have reliable methods to assess their systemic effects. In recent years, reported experimental results and theoretical interpretations have sparked an intense debate on the potential risks of nanotechnology. In this context, the interference of carbon nanomaterials with common cellular toxicity assays is commonly underrated. Malina et al. developed a new approach using a specific strategy that successfully overcame all types of interference and led to reliable results in LIVE/DEAD assays. This newly developed procedure will likely find application for all in vitro flow cytometry assays of carbon nanomaterials of any class [8]. Besides the nanoparticle surface, its shape and size could drastically influence both beneficial and detrimental properties. Regarding highly anisotropic nanoparticles, Musielak and colleagues investigated the applicability of nanorods as radiation sensitizers in prostate cancer cells. Although nanorods have the potential to increase the effectiveness of radiation in photoelectric therapy, further studies are necessary to promote their use as first-choice tools. Indeed, nanorods affected the physiology of both normal and cancer cells and the results of cellular proliferation after irradiation were ambiguous [9]. Boron nitride (BN) nanomaterials have been increasingly investigated for possible applications in various fields including biomedical, pharmaceutical, and energy industries due to their peculiar physico-chemical properties. Domi et al. assessed two commercial two-dimensional (2D) disk-like shaped BNs, namely BN-nanopowder (BN-PW) and BN-nanoplatelets (BN-PL), with a diameter of 100–150 nm and 200–300 nm, respectively. Their potential toxicity was investigated using adenocarcinoma human alveolar basal epithelial cells (A549 cells) and the unicellular fungus Saccharomyces cerevisiae, as human and environmental eukaryotic models respectively, employing in vitro assays. In both cases, cellular viability assays and reactive oxygen species determinations where performed. Even at the highest concentration and exposure time, no major cytotoxicity indicators were observed in human cells and yeast, used as target models. The results obtained in this study provided novel insights into the safety of 2D BN nanomaterials, indicating no significant differences in the toxicological potential of similar commercial products with a distinct lateral size [10]. In conclusion, this collection of articles outlines abiotic-abiotic interactions in nanomaterials and their consequent beneficial or detrimental effects, and how these interactions follow a complex scheme involving a combination of different factors, including functional groups, shape, as well as surface topography. The “Bio-Nano Interactions” special collection contributes to the newborn awareness on the need of enlightening the factors regulating the mysterious relationships between nano-surfaces recognition, bio-envelopes, and cell responses. It is a firm opinion of this author that expanding the research horizon of nanomedicine behind the current and traditional vision of theranostics as application of conventional tools, will eventually bring an enrichment to nanomedicine.
